# Urinary continence outcomes of four years of follow-up and predictors of early and late urinary continence in patients undergoing robot-assisted radical prostatectomy

**DOI:** 10.1186/s12894-020-00601-w

**Published:** 2020-03-18

**Authors:** Xing Li, Huan Zhang, Zhuo Jia, Yunpeng Wang, Yong Song, Limin Liao, Xu Zhang

**Affiliations:** 1 Department of Urology, Medical College of People’s Liberation Army, Chinese People’s Liberation Army General Hospital, No. 28 Fuxing Road, Haidian District, Beijing, 100853 China; 2 Department of Urology, Rehabilitation School of Capital Medical University, China Rehabilitation Research Center, No. 10 Jiaomen North Road, Fengtai District, Beijing, 100068 China

**Keywords:** Prostate cancer, Prostatectomy, Robotics, Urinary incontinence, Continence

## Abstract

**Background:**

The robot-assisted radical prostatectomy (RARP) has been widely applied in recent years; however, only a few studies are reported about long-term urinary continence after surgery. The present study aimed to examine the outcomes of continence rates (CRs) and determine the risk and protective factors of urinary continence in patients with prostate cancer (PCa) undergoing RARP.

**Methods:**

This retrospective study included 650 patients treated with RARP with perioperative data and at least one year of follow-up from September 2009 to November 2017. Also, the preoperative, intraoperative, and postoperative parameters of the patients were analyzed. Continence was defined as no pad use. Early and late continence was defined as the return of urinary continence within 3 months and beyond 12 months post-surgery, respectively. CRs were examined from 1 to 48 months postoperatively. Logistic regression analysis evaluated the association between the predictive factors and urinary continence in the early and late stages.

**Results:**

No significant difference was detected in the CR from 12 to 48 months postoperatively (*P* = 0.766). Logistic regression analysis proved that pelvic lymph node dissection (PLND) was a significant risk factor of urinary continence at 1 month. Nerve-sparing (NS) was a significant protective factor of urinary continence at 1, 3, and 6 months. Advanced age was an independent risk factor of urinary continence at 6, 12, and 24 months. Other variables were not statistically significant predictors of urinary continence.

**Conclusions:**

The current results demonstrated that CR gradually improved with time within 1 year and stabilized 1 year after the surgery. PLND, NS, and age were significant determinants of continence in the early and late stages, respectively. These parameters could be used for preoperative identification of patients at high risk and counseling about postoperative expectations for urinary continence.

## Background

Despite advances in surgical technique and methodology, postprostatectomy urinary incontinence (UI) remains a significant adverse event that leads to decreased quality of life [1, 2]. Several factors are involved in the recovery of urinary continence after radical prostatectomy (RP) [3, 4]. Since patients have learned about this disease from the media, it is necessary to predict the recovery of urinary continence early in order to minimize the patients’ concerns and embarrassment [5].

The technical progress has facilitated the increasing use of robot-assisted radical prostatectomy (RARP) worldwide [6]. Because the procedure can induce different degrees of damage to the bladder and urethra, UI is inevitable despite the assistance of robotic systems [7, 8]. This in turn, places physical and psychosocial burden on patients.

Reportedly, urinary continence improves only slightly in the 12 months after the surgery [2]. Hence, previous reports mainly addressed continence rates (CRs) and potential predictors within 1 year post-RARP. However, continence may continue to develop at 2 years post-surgery [7, 9, 10]. Limited data are available for longer follow-up with urinary continence after 24–48 months. In addition, a series of studies reported outcomes at 1 year after surgery, and most of them included a small number of patients (< 200). Moreover, the follow-up period was discontinuous or incomplete. For example, Yanagiuchi et al. examined and identified the outcomes at 1 and 3 months post-RARP, while Olgin et al. and Haga et al. analyzed and evaluated the outcomes at 3 and 12 months after the surgery [8, 11, 12]. In addition, Honda et al. reported continuous outcomes from 1 to 6 months postoperatively [13].

To the best of our knowledge, continuous follow-up data from 1 to 48 months on CRs and predictive factors for urinary continence after RARP with a high number of patients have not been collected. Therefore, in this retrospective study, we examined the outcomes of CRs at 1, 3, 6, 12, 24, 36, and 48 months after the surgery and determined the risk and protective factors of urinary continence.

## Methods

**Fig. 1:**
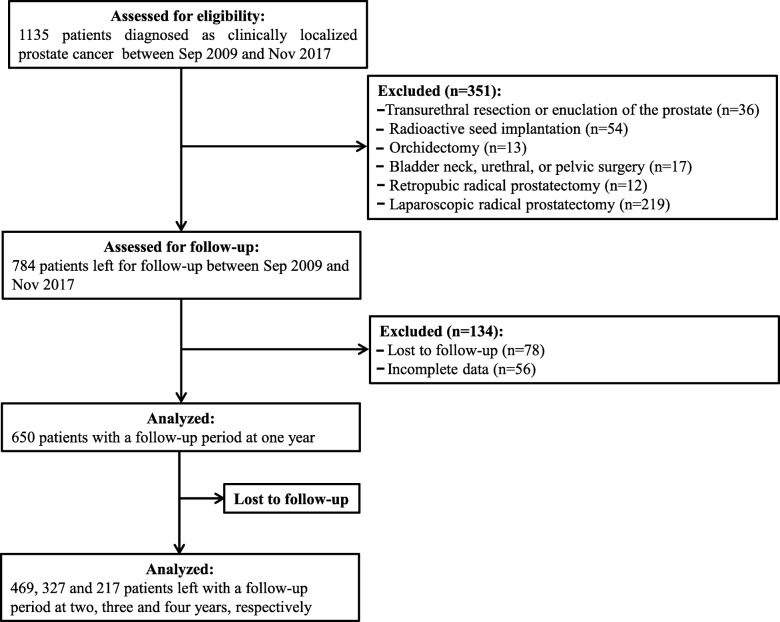
Study flow diagram

Patients diagnosed with clinically localized prostate cancer and received treatment from September 2009 to November 2017 at our institution were studied in a retrospective manner. Only patients who completed the outpatient visits or telephonic interviews for at least one year were enrolled. Patients with incomplete data and those unavailable for follow-up of continence were excluded from the study. Patients who underwent preoperative transurethral resection, enucleation of the prostate, radioactive seed implantation, orchidectomy, bladder neck, urethral, or pelvic surgery, and retropubic radical prostatectomy (RRP) or laparoscopic radical prostatectomy (LRP) were also excluded. Finally, a total of 650 eligible patients were analyzed. The schematic is illustrated in Fig. 1.

Data were collected from cases performed by three surgeons. RARP was performed by a transperitoneal approach. Nerve-sparing (NS) procedures were attempted in all potent patients at a clinical stage of T1 or T2, prostate-specific antigen (PSA) ≤10 ng/mL, and Gleason score ≤ 7. To preserve the urethral length, it was performed proximally close to the prostate while dissecting the urethra. Pelvic lymph node dissection (PLND) was performed selectively to collect samples in intermediate- and high-risk patients [14]. Urethrovesical anastomosis was carried out using 2–0 monofilament sutures with a 5/8 needle. Typically, a catheter was placed and removed at 3 weeks after the surgery.

Clinical data on the demographic characteristics and surgery-related variables were retrieved from the patient’s medical records. Together with the follow-up records, the data were assimilated into a database in this retrospective study. The factors assessed included age at the operation, body mass index (BMI), comorbidities such as hypertension (HP), diabetes mellitus (DM), coronary heart disease (CHD), and cerebrovascular diseases (CD), serum PSA level, prostate volume (PV), biopsy-determined Gleason score, clinical stage, operation time (OT), NS and PLND during surgery, and the duration of indwelling catheter (DIC) after the surgery. UI was defined as any leakage of urine after the surgery. Early and late continence was defined as the return of urinary continence within 3 months and beyond 12 months after surgery, respectively [12].

### Statistical analysis

SPSS version 19.0 (SPSS, Inc., Chicago, IL, USA) was used for statistical analysis. Data are reported as mean ± standard deviation or percentage. The chi-square test was used to compare the CRs at 12, 24, 36, and 48 months. The independent sample t-test or chi-square test was used to compare the predictive factors for urinary continence after RARP. All the above factors that might affect urinary continence were included in the univariate and multivariate logistic regression analysis to identify the risk and protective factors. *P* < 0.05 was considered statistically significant.

## Results

CRs at 1, 3, 6, 12, 24, 36, and 48 months post-surgery were 40.62, 60.92, 71.38, 78.77, 79.96, 79.51, and 76.50%, respectively (Table 1), which improved gradually within one year. The comparison of CRs at 12 and 48 months did not reveal any significant differences in the continence outcomes during the 4-year follow-up (*P* = 0.766). Consequently, CR was found to be stabilized 1 year after the operation.

**Table 1:** Continence data from 1 to 48 months after RARP

	No. continence (%)
1 month (*N* = 650)	264 (40.62%)
3 months (*N* = 650)	396 (60.92%)
6 months (*N* = 650)	464 (71.38%)
12 months (*N* = 650)	512 (78.77%)
24 months (*N* = 469)	375 (79.96%)
36 months (*N* = 327)	260 (79.51%)
48 months (*N* = 217)	166 (76.50%)

*Abbreviation*: *RARP* Robot-assisted radical prostatectomy

Furthermore, the demographic data and baseline clinical characteristics of the two groups (continence and incontinence groups) at 1 and 48 months were compared (Tables 2, 3, and 4). Fewer patients had NS, and a large number of patients had PLND in the incontinence group than those in the continence group during the surgery at a 1-month follow-up (*P* < 0.05) (Table 2). Similarly, more patients in the continence group had intraoperative NS than those in the incontinence group, as observed at the 3-month follow-up (*P* < 0.05) (Table 2). Data from the 6- and 12-month follow-ups are presented in Table 3. At a 6-month follow-up, the continence group more commonly had a NS procedure performed (*P* < 0.05). Urinary continence at 6 and 12 months after RARP was associated with age at the time of surgery (*P* < 0.05). Data from the 24- and 48-month follow-ups are presented in Table 4. Patients in the incontinence group were older than those in the continence group at 24-month follow-up (*P* < 0.05). No significant variables were observed at the 48-month follow-up.

**Table 2:** Comparison of parameters between continence and incontinence patients at 1 and 3 months after operation

Variable	1 Month			3 Months		
	Continence (*N* = 264)	Incontinence (*N* = 386)	*P* value	Continence (*N* = 396)	Incontinence (*N* = 254)	*P* value
Age (years)	65.60 ± 7.04	65.63 ± 7.94	0.959	65.20 ± 7.32	66.27 ± 7.94	0.080
BMI (kg/m^2^)	24.85 ± 2.75	25.14 ± 2.98	0.209	25.01 ± 2.82	25.02 ± 3.01	0.964
Comorbidities, no (%)						
HP	77 (29.17%)	141 (36.53%)	0.051	122 (30.81%)	96 (37.80%)	0.066
DM	38 (14.39%)	77 (16.06%)	0.563	64 (16.16%)	36 (14.17%)	0.493
CHD	20 (7.58%)	43 (11.14%)	0.131	38 (9.60%)	25 (9.84%)	0.917
CD	9 (3.41%)	11 (2.85%)	0.685	10 (2.53%)	10 (3.94%)	0.309
PSA (ng/mL)	27.65 ± 32.92	32.11 ± 36.08	0.109	28.65 ± 32.17	32.86 ± 38.64	0.133
PV (mL)	37.73 ± 20.20	35.80 ± 18.58	0.211	37.15 ± 19.34	35.70 ± 19.15	0.348
Gleason score, no (%)			0.475			0.654
≤6	79 (29.92%)	114 (29.53%)		117 (29.55%)	76 (29.92%)	
7	95 (35.98%)	160 (41.45%)		151 (38.13%)	104 (40.94%)	
≥8	78 (29.55%)	97 (25.13%)		113 (28.54%)	62 (24.41%)	
Unknown	12 (4.55%)	15 (3.89%)		15 (3.78%)	12 (4.73%)	
Clinical T stage, no (%)			0.962			0.550
T1a,b	1 (0.37%)	1 (0.26%)		1 (0.25%)	1 (0.39%)	
T1c	46 (17.42%)	68 (17.62%)		69 (17.42%)	45 (17.72%)	
T2a,b	102 (38.64%)	139 (36.01%)		157 (39.65%)	84 (33.07%)	
T2c	103 (39.02%)	155 (40.16%)		149 (37.63%)	109 (42.91%)	
T3a	1 (0.37%)	2 (0.51%)		1 (0.25%)	2 (0.79%)	
T3b	11 (4.17%)	21 (5.44%)		19 (4.80%)	13 (5.12%)	
OT (mins)	151.61 ± 42.16	153.81 ± 48.78	0.551	151.02 ± 41.76	155.86 ± 52.30	0.192
NS, no (%)	73 (27.65%)	48 (12.44%)	0.000	87 (21.97%)	34 (13.39%)	0.045
PLND, no (%)	121 (45.83%)	217 (56.22%)	0.009	204 (51.52%)	134 (52.76%)	0.757
DIC (days)	21.22 ± 10.83	21.02 ± 10.09	0.810	21.28 ± 11.46	20.84 ± 8.46	0.601

*Abbreviations*: *BMI* Body mass index, *CHD* Coronary heart disease, *CD* Cerebrovascular diseases, *DM* Diabetes mellitus, *DIC* Duration of indwelling catheter, *HP* Hypertension, *NS* Nerve-sparing, *OT* Operation time, *PSA* Prostate-specific antigen, *PV* Prostate volume, *PLND* Pelvic lymph node dissection

**Table 3:** Comparison of parameters between continence and incontinence patients at 6 and 12 months after operation

Variable	6 Months			12 Months		
	Continence (*N* = 464)	Incontinence (*N* = 186)	*P* value	Continence (*N* = 512)	Incontinence (*N* = 138)	*P* value
Age (years)	65.17 ± 7.33	66.73 ± 8.08	0.018	65.23 ± 7.36	67.06 ± 8.22	0.012
BMI (kg/m^2^)	25.05 ± 2.78	24.93 ± 3.14	0.631	25.07 ± 2.84	24.82 ± 3.06	0.362
Comorbidities, no (%)						
HP	150 (32.33%)	68 (36.56%)	0.302	172 (33.59%)	46 (33.33%)	0.954
DM	73 (15.73%)	27 (14.52%)	0.698	80 (15.63%)	20 (14.49%)	0.744
CHD	41 (8.84%)	22 (11.83%)	0.244	46 (8.98%)	17 (12.32%)	0.240
CD	12 (2.59%)	8 (4.30%)	0.253	13 (2.54%)	7 (5.07%)	0.126
PSA (ng/mL)	29.15 ± 33.12	33.16 ± 38.85	0.185	29.43 ± 32.66	32.51 ± 42.07	0.223
PV (mL)	37.14 ± 19.11	35.20 ± 19.63	0.245	36.95 ± 18.75	35.24 ± 21.10	0.354
Gleason score, no (%)			0.251			0.257
≤6	137 (29.53%)	56 (30.11%)		153 (29.88%)	40 (28.99%)	
7	178 (38.36%)	77 (41.40%)		197 (38.48%)	58 (42.03%)	
≥8	133 (28.66%)	42 (22.58%)		144 (28.13%)	31 (22.46%)	
Unknown	16 (3.45%)	11 (5.91%)		18 (3.51%)	9 (6.52%)	
Clinical T stage, no (%)			0.659			0.918
T1a,b	2 (0.43%)	0		2 (0.38%)	0	
T1c	78 (16.81%)	36 (19.35%)		88 (17.19%)	26 (18.84%)	
T2a,b	178 (38.36%)	63 (33.87%)		193 (37.70%)	48 (34.78%)	
T2c	179 (38.58%)	79 (42.47%)		201 (39.26%)	57 (41.30%)	
T3a	2 (0.43%)	1 (0.55%)		2 (0.38%)	1 (0.73%)	
T3b	25 (5.39%)	7 (3.76%)		26 (5.08%)	6 (4.35%)	
OT (mins)	152.42 ± 44.08	154.15 ± 51.17	0.666	151.66 ± 43.98	157.57 ± 53.49	0.182
NS, no (%)	97 (20.91%)	24 (12.90%)	0.018	103 (20.12%)	18 (13.04%)	0.058
PLND, no (%)	241 (51.94%)	97 (52.15%)	0.961	267 (52.15%)	71 (51.45%)	0.884
DIC (days)	21.34 ± 11.20	20.51 ± 8.00	0.356	21.37 ± 10.96	20.13 ± 7.83	0.215

*Abbreviations*: *BMI* Body mass index, *CHD* Coronary heart disease, *CD* Cerebrovascular diseases, *DM* Diabetes mellitus, *DIC* Duration of indwelling catheter, *HP* Hypertension, *NS* Nerve-sparing, *OT* Operation time, *PSA* Prostate-specific antigen, *PV* Prostate volume, *PLND* Pelvic lymph node dissection

**Table 4:** Comparison of parameters between continence and incontinence patients at 24 and 48 months after operation

Variable	24 Months			48 Months		
	Continence (*N* = 375)	Incontinence (*N* = 94)	*P* value	Continence (*N* = 166)	Incontinence (*N* = 51)	*P* value
Age (years)	63.93 ± 8.48	65.82 ± 7.62	0.016	65.70 ± 7.78	66.16 ± 7.97	0.719
BMI (kg/m^2^)	25.16 ± 2.91	24.71 ± 3.05	0.186	25.21 ± 2.89	25.30 ± 2.61	0.832
Comorbidities, no (%)						
HP	138 (36.80%)	27 (28.72%)	0.143	53 (31.93%)	20 (39.22%)	0.335
DM	60 (16.00%)	11 (11.70%)	0.299	25 (15.06%)	13 (25.49%)	0.087
CHD	40 (10.67%)	6 (6.38%)	0.212	21 (12.65%)	6 (11.76%)	0.867
CD	11 (2.93%)	4 (4.26%)	0.515	5 (3.01%)	2 (3.92%)	0.748
PSA (ng/mL)	30.00 ± 34.03	33.77 ± 35.59	0.343	30.57 ± 40.59	35.60 ± 38.12	0.433
PV (mL)	35.97 ± 19.66	34.72 ± 15.95	0.567	34.50 ± 18.85	40.11 ± 20.82	0.071
Gleason score, no (%)			0.336			0.501
≤6	109 (29.07%)	29 (30.85%)		49 (29.52%)	20 (39.22%)	
7	151 (40.27%)	42 (44.68%)		63 (37.95%)	19 (37.25%)	
≥8	103 (27.47%)	18 (19.15%)		47 (28.31%)	11 (21.57%)	
Unknown	12 (3.19%)	5 (5.32%)		7 (4.22%)	1 (1.96%)	
Clinical T stage, no (%)			0.806			0.356
T1a,b	1 (0.27%)	0		0	0	
T1c	68 (18.13%)	15 (15.96%)		32 (19.28%)	8 (15.69%)	
T2a,b	133 (35.47%)	39 (41.49%)		54 (32.53%)	11 (21.57%)	
T2c	152 (40.53%)	34 (36.17%)		70 (42.17%)	29 (56.86%)	
T3a	2 (0.53%)	0		2 (1.20%)	0	
T3b	19 (5.07%)	6 (6.38%)		8 (4.82%)	3 (5.88%)	
OT (mins)	155.19 ± 48.98	146.79 ± 42.32	0.128	154.39 ± 49.88	152.14 ± 53.29	0.782
NS, no (%)	65 (17.33%)	12 (12.77%)	0.285	20 (12.05%)	8 (15.69%)	0.498
PLND, no (%)	204 (54.40%)	52 (55.32%)	0.873	91 (54.82%)	33 (64.71%)	0.212
DIC (days)	21.27 ± 11.21	19.94 ± 5.08	0.260	21.14 ± 12.23	20.20 ± 7.93	0.603

*Abbreviations*: *BMI* Body mass index, *CHD* Coronary heart disease, *CD* Cerebrovascular diseases, *DM* Diabetes mellitus, *DIC* Duration of indwelling catheter, *HP* Hypertension, *NS* Nerve-sparing, *OT* Operation time, *PSA* Prostate-specific antigen, *PV* Prostate volume, *PLND* Pelvic lymph node dissection

Univariate and multivariate associations between urinary continence and predictive factors are shown in Tables 5, 6, and 7. In the univariate logistic analysis, PLND was associated with urinary continence at 1-month post-surgery (*P* = 0.009). In the multivariate logistic analysis, PLND was a significant independent risk factor of early urinary continence at 1 month (odds ratio (OR): 1.535, 95% confidence interval (CI): 1.079–2.184, *P* = 0.017; Table 5).

**Table 5:** Univariable and multivariable regression analysis for predictors of continence 1 and 3 months

Predictors	1 Month				3 Months			
	Univariable		Multivariable		Univariable		Multivariable	
	OR, 95% CI	P value	OR, 95% CI	*P* value	OR, 95% CI	*P* value	OR, 95% CI	*P* value
Age	1.001, 0.980–1.021	0.959	1.001, 0.979–1.023	0.940	1.019, 0.998–1.041	0.080	1.018, 0.996–1.041	0.113
BMI	1.036, 0.981–1.094	0.209	1.023, 0.963–1.086	0.460	1.001, 0.948–1.057	0.964	0.998, 0.941–1.059	0.947
HP	1.398, 0.998–1.957	0.051	1.376, 0.947–1.999	0.094	1.365, 0.980–1.901	0.066	1.404, 0.978–2.015	0.066
DM	1.138, 0.734–1.764	0.563	0.999, 0.626–1.595	0.998	0.857, 0.550–1.334	0.493	0.748, 0.470–1.190	0.220
CHD	1.529, 0.878–2.665	0.134	1.476, 0.817–2.688	0.197	1.028, 0.605–1.750	0.917	0.885, 0.508–1.542	0.665
CD	0.831, 0.340–2.034	0.685	0.739, 0.289–1.889	0.528	1.582, 0.649–3.856	0.313	1.456, 0.582–3.640	0.422
PSA	1.004, 0.999–1.009	0.114	1.000, 0.995–1.005	0.985	1.003, 0.999–1.008	0.138	1.002, 0.997–1.006	0.501
PV	0.995, 0.987–1.003	0.212	0.994, 0.986–1.002	0.159	0.996, 0.988–1.004	0.348	0.995, 0.987–1.004	0.262
Gleason score	0.929, 0.774–1.116	0.433	0.801, 0.654–0.981	0.332	0.965, 0.802–1.160	0.702	0.926, 0.758–1.131	0.449
Clinical stage	1.060, 0.899–1.250	0.487	1.013, 0.850–1.208	0.883	1.077, 0.913–1.270	0.379	1.071, 0.902–1.273	0.434
OT	1.001, 0.998–1.004	0.551	1.000, 0.996–1.003	0.931	1.002, 0.999–1.006	0.193	1.002, 0.998–1.005	0.281
NS	0.372, 0.248–0.557	0.000	0.360, 0.231–0.561	0.000	0.549, 0.356–0.846	0.007	0.546, 0.342–0.872	0.011
PLND	1.517, 1.018–2.079	0.009	1.535, 1.079–2.184	0.017	1.051, 0.767–1.441	0.757	0.978, 0.690–1.386	0.901
DIC	0.998, 0.983–1.013	0.809	0.999, 0.983–1.014	0.869	0.996, 0.980–1.012	0.602	0.997, 0.981–1.013	0.713

*Abbreviations*: *BMI* Body mass index, *CHD* Coronary heart disease, *CD* Cerebrovascular diseases, *DM* Diabetes mellitus, *DIC* Duration of indwelling catheter, *HP* Hypertension, *NS* Nerve-sparing, *OT* Operation time, *PSA* Prostate-specific antigen, *PV* Prostate volume, *PLND* Pelvic lymph node dissection

**Table 6:** Univariable and multivariable regression analysis for predictors of continence 6 and 12 months

Predictors	6 Months				12 Months			
	Univariable		Multivariable		Univariable		Multivariable	
	OR, 95% CI	*P* value	OR, 95% CI	*P* value	OR, 95% CI	*P* value	OR, 95% CI	*P* value
Age	1.028, 1.005–1.052	0.019	1.026, 1.001–1.051	0.038	1.034, 1.007–1.061	0.012	1.030, 1.002–1.058	0.035
BMI	0.986, 0.929–1.046	0.630	0.993, 0.932–1.058	0.827	0.970, 0.908–1.036	0.362	0.983, 0.916–1.054	0.626
HP	1.206, 0.845–1.723	0.302	1.179, 0.800–1.737	0.404	0.988, 0.663–1.472	0.954	0.937, 0.606–1.448	0.768
DM	0.910, 0.564–1.468	0.698	0.834, 0.506–1.375	0.477	0.915, 0.538–1.556	0.744	0.873, 0.501–1.521	0.632
CHD	1.384, 0.800–2.395	0.246	1.190, 0.672–2.109	0.551	1.423, 0.788–2.571	0.242	1.275, 0.686–2.371	0.443
CD	1.693, 0.681–4.211	0.258	1.583, 0.617–4.064	0.339	2.051, 0.802–5.244	0.134	2.144, 0.806–5.072	0.126
PSA	1.003, 0.998–1.008	0.190	1.001, 0.996–1.006	0.614	1.003, 0.998–1.008	0.227	1.002, 0.996–1.007	0.517
PV	0.995, 0.985–1.004	0.245	0.995, 0.985–1.004	0.288	0.995, 0.985–1.005	0.354	0.995, 0.985–1.006	0.393
Gleason score	0.976, 0.800–1.192	0.815	0.946, 0.764–1.173	0.614	1.017, 0.816–1.267	0.879	0.998, 0.788–1.264	0.989
Clinical stage	0.973, 0.814–1.164	0.767	0.972, 0.807–1.171	0.765	0.996, 0.818–1.214	0.970	0.991, 0.807–1.218	0.932
OT	1.001, 0.997–1.004	0.665	1.001, 0.997–1.005	0.720	1.003, 0.999–1.007	0.182	1.003, 0.999–1.007	0.177
NS	0.561, 0.346–0.909	0.019	0.545, 0.324–0.915	0.022	0.596, 0.347–1.023	0.060	0.609, 0.342–1.086	0.093
PLND	1.008, 0.717–1.418	0.961	0.993, 0.683–1.444	0.971	0.972, 0.668–1.416	0.884	0.914, 0.606–1.380	0.670
DIC	0.991, 0.973–1.010	0.359	0.993, 0.975–1.012	0.474	0.986, 0.964–1.008	0.215	0.988, 0.966–1.011	0.304

*Abbreviations*: *BMI* Body mass index, *CHD* Coronary heart disease, *CD* Cerebrovascular diseases, *DM* Diabetes mellitus, *DIC* Duration of indwelling catheter, *HP* Hypertension, *NS* Nerve-sparing, *OT* Operation time, *PSA* Prostate-specific antigen, *PV* Prostate volume, *PLND* Pelvic lymph node dissection

**Table 7:** Univariable and multivariable regression analysis for predictors of continence 24 and 48 months

Predictors	24 Months				48 Months			
	Univariable		Multivariable		Univariable		Multivariable	
	OR, 95% CI	*P* value	OR, 95% CI	*P* value	OR, 95% CI	*P* value	OR, 95% CI	*P* value
Age	1.970, 1.943–1.998	0.017	1.968, 1.939–1.997	0.038	1.008, 0.967–1.049	0.717	1.009, 0.964–1.057	0.691
BMI	0.949, 0.877–1.026	0.186	0.956, 0.877–1.042	0.310	1.012, 0.906–1.131	0.831	0.999, 0.880–1.136	0.992
HP	0.692, 0.422–1.134	0.144	0.810, 0.474–1.384	0.441	1.376, 0.718–2.635	0.336	1.484, 0.708–3.109	0.296
DM	0.696, 0.350–1.383	0.301	0.779, 0.382–1.589	0.492	1.929, 0.902–4.125	0.090	1.803, 0.803–4.051	0.153
CHD	0.571, 0.235–1.390	0.217	0.642, 0.255–1.617	0.347	0.921, 0.350–2.421	0.867	0.847, 0.293–2.448	0.759
CD	1.471, 0.458–4.726	0.517	1.816, 0.535–6.160	0.339	1.314, 0.247–6.987	0.749	1.003, 0.158–6.389	0.997
PSA	1.003, 0.997–1.009	0.346	1.003, 0.996–1.009	0.419	1.003, 0.996–1.010	0.437	1.002, 0.995–1.010	0.530
PV	0.996, 0.984–1.009	0.567	0.996, 0.983–1.009	0.580	1.014, 0.999–1.029	0.077	1.014, 0.998–1.031	0.093
Gleason score	0.919, 0.699–1.207	0.542	0.892, 0.659–1.208	0.461	0.744, 0.509–1.089	0.128	0.691, 0.452–1.058	0.089
Clinical stage	1.013, 0.802–1.280	0.912	1.054, 0.824–1.348	0.677	1.222, 0.886–1.684	0.221	1.189, 0.835–1.692	0.337
OT	0.996, 0.991–1.001	0.128	0.997, 0.991–1.002	0.210	0.999, 0.993–1.005	0.781	0.998, 0.991–1.005	0.492
NS	0.698, 0.360–1.353	0.287	0.719, 0.352–1.472	0.367	1.358, 0.559–3.299	0.499	1.163, 0.427–3.167	0.767
PLND	1.038, 0.659–1.635	0.873	1.145, 0.690–1.902	0.600	1.511, 0.788–2.896	0.214	1.720, 0.828–3.573	0.146
DIC	0.983, 0.955–1.012	0.257	0.980, 0.949–1.013	0.228	0.991, 0.958–1.026	0.605	0.989, 0.950–1.030	0.604

*Abbreviations*: *BMI* Body mass index, *CHD* Coronary heart disease, *CD* Cerebrovascular diseases, *DM* Diabetes mellitus, *DIC* Duration of indwelling catheter, *HP* Hypertension, *NS* Nerve-sparing, *OT* Operation time, *PSA* Prostate-specific antigen, *PV* Prostate volume, *PLND* Pelvic lymph node dissection

Univariate logistic analysis showed that NS was associated with urinary continence at 1 month (OR: 0.372, 95% CI: 0.248–0.557, *P* < 0.001), 3 months (OR: 0.549, 95% CI: 0.356–0.846, *P* = 0.007), and 6 months (OR: 0.561, 95% CI: 0.346–0.909, *P* = 0.019) (Tables 5 and 6). In multivariate logistic analysis, NS was a significant independent protective factor of urinary continence at 1 month (OR: 0.360, 95% CI: 0.231–0.561, *P* < 0.001), 3 months (OR: 0.546, 95% CI: 0.342–0.872, *P* = 0.011), and 6 months (OR: 0.545, 95% CI: 0.324–0.915, *P* = 0.022) (Tables 5 and 6).

Univariate logistic analysis showed that younger age was associated with urinary continence at 6 months (OR: 1.028, 95% CI: 1.005–1.052, *P* = 0.019), 12 months (OR: 1.034, 95% CI: 1.007–1.061, *P* = 0.012), and 24 months (OR: 1.970, 95% CI: 1.943–1.998, *P* = 0.017) (Tables 6 and 7). In multivariate logistic analysis, advanced age was a significant independent risk factor of urinary continence at 6 months (OR: 1.026, 95% CI: 1.001–1.051, *P* = 0.038), 12 months (OR: 1.030, 95% CI: 1.002–1.058, *P* = 0.035), and 24 months (OR: 1.968, 95% CI: 1.939–1.997, *P* = 0.038) (Tables 6 and 7). Other variables mentioned above were not statistically significant predictors. Also, no significant predictors of late urinary continence were detected at 48 months (Table 7).

## Discussion

Reportedly, the urinary continence is stable up to 12 months after RP [2]. Hence, previous studies have addressed CRs within 1 year post-RARP. Only little data are available at more than 24 months follow-up for urinary continence. In the current study, the CRs were 78.77, 79.96, 79.51, and 76.50% at 1, 2, 3, and 4 years after the surgery, respectively (Table 1). No significant differences were observed in the continence outcomes during the 4-year follow-up. Our results certified that one year after RARP was the stable continence period [15]. Few studies have evaluated the CRs after 12 months. Shao et al. reported that CR was 89.4% at 24 months after RARP, while Xylinas et al. reported that the 24-month urinary continence rate was 88% based on the no-pad definition [7, 9]. Murphy et al. reported a 36-month urinary continence rate of 94.7% using the no-pad or safety pad definition [10]. Mandel et al. reported that CRs were 89.5 and 90.9% at 24 and 36 months after the surgery, respectively [16]. The CR of the current study was 76.50% at 48 months after RARP, which has been the longest follow-up on the topic to date.

A large number of studies have evaluated the predictors of urinary continence within 1 year after surgery. These studies either included a relatively small number of patients or had a discontinuous follow-up. To the best of our knowledge, this is the first study to evaluate the predictors of continence from 1 to 48 months after RARP in a large sample.

In the current cohort, the CRs were 40.62, 60.92, 71.38, and 78.77% at 1, 3, 6 and 12 months after RARP, respectively (Table 1). These results were in agreement with those from the recent study by Honda et al. that revealed CRs at 1, 3, and 6 months as 40.7, 63.0, and 73.1%, respectively [13]. The definition of postoperative urinary continence varied among several studies. Hitherto, there is no consensus on UI post-RP [17]. Herein, we selected the most stringent definition of incontinence: any leakage of urine. Reportedly, the continence rate one year after RARP is 69–97% [1, 18]. The overall continence rate in this study was 78.77% at 12 months, without the usage of any pad. Although it is not excellent, it is within the average range.

To identify the predictive factors, urinary continence was divided into two categories: early continence (< 3 months) and late continence (> 12 months) [12].

In general, PLND was selectively performed for sampling purposes in intermediate- and high-risk patients [14]. One month after the surgery, PLND in the continence group occurred in 121 patients (45.83%), while it occurred in 217 patients in the incontinence group (56.22%) (*P* < 0.05). Three months post-surgery, no significant differences were detected in both groups. The logistic analysis showed that PLND was a significant independent risk factor of early urinary continence at 1 month. Patients who had undergone PLND during surgery had a high risk of UI. Lymphadenectomy may give rise to transient damage to nerve vessel bundles (NVBs), which affected the recovery of urinary continence. However, with the recovery of body function, this impact declined gradually.

The current logistic analysis showed that NS was a significant independent protective factor of urinary continence at 1, 3, and 6 months (Tables 5 and 6). These findings were in line with those from the study by Reeves et al., which found that avoiding damage to the nerves around the prostate improves urinary continence in the first 6 months after the surgery [19]. Michl et al. investigated long-term CRs (12 months) after RP and found a significant difference between the NS and non-NS groups [20]. The studies by Kadono et al. and Steineck et al. also indicated that NS is associated with urinary continence in the long-term [15, 21]. The bias in the studies used for the analysis might have influenced the results. However, all the studies showed that NS during the surgery produced satisfactory postoperative continence outcomes.

Multiple studies have demonstrated that age is an independent risk factor for the return of continence at 1–12 months after RARP. Lavigueur-Blouin et al. evaluated the early continence after RARP [22]. It showed that advanced age was an independent predictor at 1 month. Kim et al. demonstrated that younger men could have an early recurrence of continence 3 months after RARP [23]. Greco et al. compared the continence outcomes of RARP in older men to those of the younger men [24]. The study showed that CRs at 1, 3, and 12 months were similar in the two groups; however, the older group had a significantly lower continence rate at 6 months. The results of these studies might partially be in agreement with those of our study. Shikanov et al. demonstrated that age is a predictor of continence return at 12 months after RARP, which is partially in accordance with the current results [25]. Our results show that advanced age is a significant risk factor of continence at 6, 12, and 24 months after the surgery (Tables 6 and 7). Men of advanced age had a high risk of UI. Older men have poor endothelial dysfunction, which affects the vascular supply of the NVBs. In addition, it is difficult to perform pelvic floor exercises (PLE) due to an age-related decrease in the mass of the skeletal muscle and neuronal plasticity [26]. These conditions might affect functional outcomes.

Interestingly, the optimal time to remove the indwelling catheter has not yet been determined. Conventionally, the urinary catheter was removed between 10 and 21 days postoperatively [27]. Typically, the catheter is removed 3 weeks after the operation in our center in order to ensure the healing of anastomosis. However, most patients returned home after the operation, and the indwelling catheter was removed at the local clinic. This leads to the inconsistency of catheter removal and the duration of indwelling catheter might be longer than expected.

### Limitations

Nevertheless, the present study had some limitations. First, this was a retrospective study from a single institution, and surgeries were not performed by a single surgeon. Second, we did not analyze all variables due to the undocumented surgical steps of the procedure, variations in surgical experience, and differences in the pathological reports. Third, although a stringent definition of continence was applied, the conditions were reported by the patients rather than based on a quality questionnaire. Fourth, the potential bias in selecting the patients for the procedure might influence the results. In addition, data were missing as many patients were lost to follow-up. Despite these drawbacks, this study utilized a large sample size, and the survey of postoperative urinary continence was time-continuous.

## Conclusions

The current study has shown that CR improves gradually with time within 1 year post-surgery and stabilizes after one more year. PLND, NS, and age are the significant determinants of continence in the early and late stages, respectively. Thus, these parameters could be used to identify patients at high risk for UI preoperatively and counsel them on postoperative expectations for urinary continence.

## Data Availability

All the data supporting our findings is contained in the manuscript. The datasets used and/or analysed in the current study is available from the corresponding author on reasonable request.

## Abbreviations

BMI: Body mass index
CRs: Continence rates
CHD: Coronary heart disease
CD: Cerebrovascular diseases
CI: Confidence interval
DM: Diabetes mellitus
DIC: Duration of indwelling catheter
HP: Hypertension
LRP: Laparoscopic radical prostatectomy
NS: Nerve-sparing
NVBs: Nerve vessel bundles
OT: Operation time
OR: Odds ratio
RARP: Robot-assisted radical prostatectomy
RRP: Retropubic radical prostatectomy
PSA: Prostate-specific antigen
PV: Prostate volume
PLND: Pelvic lymph node dissection
PLE: Pelvic floor exercises
RP: Radical prostatectomy
UI: Urinary incontinence
